# Postnatal Common Mental Disorders and Their Predictors in Northwest Ethiopia: A Community‐Based Cohort Study

**DOI:** 10.1155/da/2781884

**Published:** 2026-05-04

**Authors:** Helina Abebe Kurbi, Solomon Mekonnen Abebe, Netsanet Worku Mengistu, Alemayehu Teklu Toni, Tadesse Awoke Ayele

**Affiliations:** ^1^ Department of Epidemiology and Biostatistics, Institute of Public Health, College of Medicine and Health Sciences, University of Gondar, Maraki Street, Gondar, Ethiopia, uog.edu.et; ^2^ Department of Nutrition, Institute of Public Health, College of Medicine and Health Sciences, University of Gondar, Maraki Street, Gondar, Ethiopia, uog.edu.et; ^3^ Department of Pediatrics and Child Health, College of Medicine and Health Sciences, University of Gondar, Maraki Street, Gondar, Ethiopia, uog.edu.et

**Keywords:** Ethiopia, labor complications, postnatal common mental disorders, preterm births

## Abstract

**Background:**

Postnatal common mental disorders (PCMDs) are the most common complications after childbirth and are associated with many adverse effects on infant growth and development. Undiagnosed and untreated PCMDs significantly affect the health and lives of the mother, their children, and families. Although PCMDs are a significant public health concern, evidence from Ethiopia is limited. This study aimed to assess PCMDs and their predictors among postnatal women in the Dabat HDSS, Northwestern Ethiopia.

**Methods:**

A community‐based cohort study involved 872 pregnant women, who were evaluated for common mental disorders during their second and third trimesters and again two to 8 weeks postpartum. Women with a self‐reporting questionnaire‐20 (SRQ‐20) score of ≥6 were considered to have common mental disorders. A modified Poisson regression model was used to identify the independent predictors of pCMDs.

**Results:**

The prevalence and incidence of pCMDs were 16.05% (95% CI: 13.67, 18.74) and 14.02% (95% CI: 11.64, 16.79), respectively, with 3.83% of women exhibiting perinatal CMD during the study period. PCMDs are independently predicted by experiencing labor complications (IRR = 2.43, 95% CI: 1.69, 3.47), preterm birth (IRR = 1.72, 95% CI: 1.26, 2.35), antenatal CMD (IRR = 1.89, 95% CI: 1.37, 2.61), and a history of CMD before pregnancy (IRR = 2.29, 95% CI: 1.32, 3.98).

**Conclusion:**

The observed incidence and prevalence of PCMDs in Ethiopia were lower than in previous studies. Common mental disorders before and during pregnancy, preterm birth, and the presence of labor complications increase the risk of pCMDs. Early detection and treatment of mental disorders before and during pregnancy, along with interventions to reduce preterm birth and labor complications, could decrease the incidence of postnatal mental disorders.

## 1. Background

Postnatal common mental disorders (PCMDs) are the most common pregnancy and childbirth complications, causing biological, physical, social, and emotional challenges that affect women’s daily functioning and their children’s growth and development [1–3]. Postnatal mental disorders, including depression, anxiety, and somatic issues, significantly increase the global disease burden [4–6], affecting 10%–15% in high‐income countries (HICs) and over 30% of women in low‐ and middle‐income countries (LMICs) [7, 8].

The World Health Organization (WHO) estimates that ~20%–40% of women in low‐income countries develop common mental disorders during pregnancy and after childbirth [3]. Ethiopia has also been experiencing a high rate of postnatal CMD, with a reported pooled prevalence of 22% for postnatal depression [9, 10]. Furthermore, research conducted across different regions of Ethiopia indicates that the prevalence of postnatal mental disorders ranges from 18% to 33%, showing that approximately one in six women is affected [11–13].

Risk factors such as hormonal fluctuations, preexisting mental health conditions, poverty, domestic violence, low social and partner support, obstetric complications, unplanned pregnancy, poor marital relationships, infant mortality, substance use, and stigma significantly increase the prevalence of common mental disorders in the postnatal period [9–14]. Additionally, studies have shown that antenatal common mental disorders are negatively associated with unhealthy behaviors, such as missing antenatal care, low self‐care, and substance abuse, which can lead to poor birth outcomes and the development of CMD after birth [15–18].

Women with antenatal depression often continue to experience depressive symptoms after childbirth, and more than half of those with postpartum depression report episodes during or before pregnancy [19–21]. In LMICs like Ethiopia, antenatal mental health remains largely overlooked and undertreated because the primary focus is on preventing maternal deaths from obstetric complications [22, 23]. Untreated antenatal mental health issues are concerning because they are associated with postpartum mental disorders and can negatively affect child growth and development [24, 25].

Postnatal mental disorders continue to be a significant public health concern in Ethiopia, affecting child growth and development. However, the previously conducted research mostly depends on cross‐sectional, facility‐based studies, highlighting the need for a prospective research design [11, 13]. Additionally, most of those studies focus on postnatal depression but overlook the broader range of common mental disorders classified globally, and few studies have examined the incidence and prevalence of postnatal CMD [12]. Data on risk factors, including antenatal stressors such as common mental disorders before and during pregnancy, are limited. As a result, the relationships between antenatal CMD, other stressors, and postnatal CMD are not well understood. To address this research gap, a prospective cohort study was conducted to estimate the incidence and prevalence of postnatal CMD and to identify predictors using a modified Poisson regression model in the Dabat HDSS, Northwest Ethiopia.

## 2. Methods

### 2.1. Study Design

A community‐based prospective study was conducted, with pregnant women initially recruited during their second or third trimesters and reassessed in the postnatal period among women residing in the Dabat HDSS, Northwest Ethiopia, from June 2, 2022, to July 30, 2023.

### 2.2. Study Setting and Population

This research was conducted at the Dabat HDSS in Northwest Ethiopia. The University of Gondar established the Dabat HDSS site in 1996. Currently, the Dabat HDSS site includes 13 Kebeles (four urban and nine rural), over 21,000 households, and a population of 70,000 in the Dabat district. This system collects longitudinal data on vital events (births, deaths, and migrations) to estimate population‐level causes of death using the verbal autopsy method. Dabat HDSS uses health and demographic data, such as events, social and economic conditions, pregnancies, births, and disease outbreaks, to guide policies and programs aimed at improving the well‐being of the local population [26]. Women who were unable to communicate due to severe illness during pregnancy were excluded from the study.

### 2.3. Sample Size Determination

This study is part of a community‐based prospective cohort of mother‐infant dyads in the Dabat HDSS, Northwest Ethiopia, that examines the effects of perinatal CMD on birth outcomes, infant nutritional status, and developmental outcomes. The sample size was estimated using Epi‐Info Version 7 [27]. The double population formula was used with the assumption of 95% confidence level, 90% power, an exposure‐to‐nonexposure ratio of 1:2, and the prevalence of low birth weight among those free from antenatal depression of 21% [28], an effect size of 1.5, and 10% loss to follow‐up; as a result, the final sample size was estimated to be 946. A total of 872 pregnant women were recruited, of whom 810 completed follow‐ups during the postnatal period.

### 2.4. Data Collection and the Questionnaire

A face‐to‐face interview was conducted in the homes of postnatal mothers using an online Open Data Collection Kit (ODK), designed to collect and manage data in low‐income settings [29]. Twenty‐eight data collectors and seven supervisors received 3 days of training in the district’s local language, and a pretest was administered 15 days before the actual data collection. The principal investigator maintained regular contact with the team to ensure proper data collection, and the collected data were stored and uploaded via a wireless connection to the Dabat HDSS in Northwest Ethiopia.

### 2.5. Operational Definition

PCMD was assessed using the WHO Self‐Reporting 20 Questionnaire (SRQ‐20). The questionnaire includes 20 items that evaluate symptoms of depression, anxiety, panic, and somatic issues experienced in the past month, providing an overall measure of mental distress [30]. The SRQ‐20 has been prevalidated for pregnant and postnatal women in the Butajira population in Ethiopia [31]. A cutoff point of six yielded a sensitivity of 90.7% and a specificity of 80.7%. To meet the current objectives, the total score was dichotomized (SRQ‐20 ≥ 6), with a high score indicating a high level of CMD. The Cronbach’s alpha for internal consistency was (*α* = 0.82). The SRQ‐20 was selected for its prior validation in Ethiopia, including among perinatal women, and for its suitability for community screening in low‐resource settings. The CMD history was evaluated using the question: Have you previously experienced any symptoms of depression or anxiety before this pregnancy?

The WHO Multi‐Country Study of Women’s Health questionnaire was used to identify the presence of intimate partner violence. It included questions about psychological, physical, and sexual violence, and a single yes response indicated violence [32]. The Oslo Social Support Scale 3 (OSSS‐3) assesses social support during pregnancy with 3 Likert‐scale items. Scores range from 0 to 14, with below 9 indicating “poor” support and 9–14 “strong” support [33]. The “Life‐Threatening Experience Questionnaire” was used to evaluate life‐threatening experiences during pregnancy. It comprises 12 items that assess events such as death, illness, conflict, and property damage. The questionnaire was adapted to the local conditions and time frames of the current pregnancy and translated into Amharic. The scale has been rigorously tested and has shown good test–retest reliability, with Kappa values ranging from 0.61 to 0.87. Furthermore, the questionnaire possesses predictive validity and is valuable for assessing and managing life‐threatening situations during pregnancy [34].

### 2.6. Statistical Analysis and Management

The complete dataset was downloaded from the Dabat HDSS server as an Excel spreadsheet, checked for completeness, and imported into Stata version 16 (Stata Corp., USA) for further data cleaning and analysis. Descriptive analysis involved calculating the mean, median, proportion, and standard deviation. Explanatory analyses, such as cross‐tabulations and frequency distributions, were conducted to better understand the data. Chi‐squared tests were performed to assess the crude associations between the predictors and the risk of postnatal CMD.

Binomial and Poisson regression models are recommended for estimating the relative risk [35]. However, because they are known for convergence issues, problems may arise, leading to overestimation. This problem can be addressed using a robust error‐variance procedure called the modified Poisson regression model [36]. In this study, we estimated the relative risk using a generalized linear model with an identity link and a binomial distribution (log‐binomial), but we encountered convergence issues.

The incidence risk ratio was selected as the appropriate effect measure because it is less likely to be misinterpreted. Bivariate and multivariable analyses were conducted to examine the relationship between covariates and the outcome variable. Crude and adjusted relative risks, along with 95% confidence intervals, were reported for predictor variables with *p*‐values less than 0.05. Goodness‐of‐fit was assessed using the Hosmer–Lemeshow and Omnibus tests. Multicollinearity was checked by examining each predictor’s variance inflation factor (VIF). All VIF values remained below the threshold of 5, with the highest being 1.10, indicating no significant multicollinearity among the independent variables. The manuscript was prepared in accordance with the Strengthening the Reporting of Observational Studies in Epidemiology (STROBE) guidelines. The completed STROBE checklist is included as Supporting Information.

## 3. Result

A total of 824 mother‐infant dyads were followed from birth to 2 months postpartum. During the follow‐up, five participants withdrew, and nine could not be reached due to address changes. The response rate was 98.3%, resulting in a final dataset of 810 mother‐infant pairs for analysis.

### 3.1. Sociodemographic Characteristics of Participants

The sociodemographic characteristics of the study participants are presented in Table 1. The mean (SD) age of the participants was 27.7 (6.21) years, and most participants (*n* = 681, 84.07%) were responsible for household chores. The majority identified as Orthodox Christians (*n* = 776, 95.80%) and were married (*n* = 637, 78.64%). There were no significant differences in sociodemographic characteristics of the participants with and without PCMDs, except for household monthly income. A higher proportion of study participants with PCMDs had low income (18.02%) compared to those without (16.85%) (Table 1).

**Table 1:** Sociodemographic characteristics of study participants in the Dabat HDSS, Northwest Ethiopia.

Variable (category)	Postnatal CMD		Total	*p*‐Value
	Yes (*n* = 130)	No (*n* = 680)		
Women’s age at enrollment				
18–25	53 (16.01)	278 (83.99)	331	0.887
26–35	61 (15.68)	328 (84.32)	389	
≥36	16 (17.78)	74 (82.22)	90	
Monthly household income				
Low	102 (18.02)	464 (81.98)	556	0.015
Medium	13 (8.39)	142 (91.61)	155	
High	15 (16.85)	74 (83.15)	89	
Women’s Education				
Unable to read and write	72 (16.07)	376 (83.93)	448	0.304
Able to read and write	30 (19.48)	124 (80.52)	154	
Primary and above	28 (13.46)	180 (86.54)	208	
Women’s occupation				
Household duties	114 (16.74)	567 (83.26)	681	0.219
Other^a^	16 (12.40)	113 (87.60)	129	
Women’s religion				
Orthodox Christian	126 (16.24)	650 (83.76)	776	0.487
Muslim	4 (11.76)	30 (88.24)	34	
Women’s Marital Status				
Married	100 (15.70)	537 (84.30)	637	0.602
Others^b^	30 (17.34)	143 (82.66)	173	
Difficulty of accessing food in the last 3 months				
Yes	79 (15.67)	425 (84.33)	504	0.709
No	51 (16.67)	255 (83.33)	306	

Note: *p*‐Value was based on chi‐square test statistics.

^a^Government employee and self‐employee.

^b^Single, divorced, and widowed.

### 3.2. Obstetric Characteristics of Study Participants

The obstetric and behavioral characteristics of the study participants are presented in Table 2. A higher incidence of PCMDs was observed among those who self‐reported postnatal complications (*p*  < 0.001), low birth weight (*p* = 0.058), and preterm births (*p*  < 0.001). Most pregnancies were planned (73.95%), and half of the participants (50.25%) had at least one antenatal care visit (Table 2).

**Table 2:** Obstetric and behavioral characteristics of the study participants in the Dabat HDSS, Northwest Ethiopia.

Variables (category)	Postnatal CMD		Total	*p*‐Value
	Yes (*n* = 130)	No (*n* = 680)		
Pregnancy Intention				
Yes	88 (14.69)	511 (85.31)	599	0.076
No	42 (19.91)	169 (80.09)	211	
History of Abortion				
Yes	8 (11.37)	50 (86.21)	58	0.627
No	130 (16.05)	680 (83.95)	752	
Antenatal care service uptake (at least once)				
Yes	64 (15.88)	339 (84.12)	403	0.897
No	66 (16.22)	341 (83.75)	407	
Postnatal care service				
Yes	107 (15.55)	581 (84.45)	688	0.360
No	23 (18.85)	99 (81.16)	122	
Low birth weight				
Yes	17 (23.94)	54 (76.06)	73	0.058
No	113 (15.29)	626 (84.71)	739	
Preterm				
Yes	38 (28.36)	96 (71.64)	—	<0.001
No	92 (13.61)	584 (86.39)	—	
Self‐Reported Postnatal Complications				
Yes	28 (33.33)	56 (66.67)	84	<0.001
No	102 (14.05)	624 (85.95)	726	

### 3.3. Psychosocial Characteristics of the Study Participants

Table 3 presents the participants’ psychosocial characteristics. Among them, 90 individuals (11.11%) had a history of common mental disorders before pregnancy, 59 (7.28%) experienced low social support, and 385 (47.53%) reported frequently receiving support from their partners. Participants with PCMDs more often reported a history of mental disorders before pregnancy (*p* = 0.002) and during pregnancy (*p*  < 0.001) (Table 3).

**Table 3:** Psychosocial characteristics of study participants at the Dabat HDSS, Northwest Ethiopia.

Variables (category)	Postnatal CMD		Total	*p*‐Value
	Yes (130)	No (680)		
Intimate partner violence				
Yes	52 (16.51)	263 (83.49)	315	0.777
No	78 (15.76)	419 (84.24)	495	
Social Support				
Good	121 (16.11)	630 (83.89)	751	0.863
Poor	9 (15.25)	50 (84.75)	59	
Life‐threatening events				
Yes	45 (15.20)	251 (84.80)	296	0.618
No	85 (16.54)	429 (83.46)	514	
History of common mental disorders				
Yes	11 (36.67)	19 (63.33)	30	0.002
No	119 (15.26)	661 (84.74)	780	
Partner Support				
Yes	98 (15.03)	554 (84.97)	652	0.109
No	32 (20.25)	126 (79.75)	158	
Antenatal common mental disorders				
Yes	31 (29.81)	73 (70.19)	104	<0.001
No	99 (14.02)	607 (85.98)	706	
Family history of Psychiatric illness				
Yes	9 (20.93)	34 (79.07)	43	0.370
No	121 (15.78)	646 (84.22)	767	

### 3.4. Incidence and Prevalence of PCMDs

The initial screening for common mental disorders takes place during pregnancy. Later, a cohort of 810 women was rescreened for CMD during the postpartum period. Among these women, 130 (16.05%) tested positive for postnatal CMD (16.05% 95% CI: 13.67, 18.74). Ninety‐nine women developed symptoms of common mental disorders after their initial screening, indicating an incidence proportion of 14.02% (95% CI: 11.64, 16.79), and 31 women (3.83%) had persistent common mental disorders, testing positive at both screenings. Additionally, 203 women experienced a common mental disorder at least once during the screening period, resulting in a perinatal CMD rate of 25.06%.

The bivariate analysis shows that the prevalence of PCMDs was higher among study participants with low household income, who reported postnatal complications, experienced unplanned pregnancies, and had infants with low birth weight and preterm birth. Additionally, PCMDs were more common among participants who reported poor partner support and had a history of common mental disorders before and during pregnancy.

### 3.5. Predictors of the Incidence and Prevalence of PCMDs

Table 4 presents predictors of PCMDs from a multivariable modified Poisson regression model. In this model, preterm birth, the presence of postnatal complications, and a history of CMDs before and during pregnancy independently predicted the incidence of PCMDs. The risk of postnatal mental disorders was 2.43 and 1.72 times higher (IRR = 95% CI: 1.69, 3.47) and (IRR = 95% CI: 1.26, 2.35), respectively, among participants who reported postnatal complications and had preterm birth. The presence of CMDs during pregnancy increased the risk of PCMDs by 1.89 times (IRR = 95% CI: 1.37, 2.61). Women with a history of CMD before pregnancy had 2.29 times higher risk (IRR = 95% CI: 1.32, 3.98) of postnatal mental disorders compared to those without such a history (Table 4).

**Table 4:** Bivariable and multivariable modified Poisson regression models of predictors for the incidence of postnatal common mental disorders in Dabat HDSS, Northwest Ethiopia.

Variables	Postnatal CMD		CRR^a^, 95% CI	ARR^b^, 95% CI
	Yes, *N* (%)	No, *N* (%)		
Preterm birth				
Yes	38 (28.36)	96 (71.64)	2.08 (1.49, 2.89)	1.72 (1.26, 2.35)
No	92 (13.61)	584 (86.39)	1	
Postnatal complications				
Yes	28 (33.33)	56 (66.67)	2.37 (1.66, 3.37)	2.43 (1.69, 3.47)
No	102 (14.05)	624 (85.95)	1	
History of CMD before pregnancy				
Yes	11 (36.67)	19 (63.33)	2.40 (1.45, 3.95)	2.29 (1.32, 3.98)
No	119 (15.26)	661 (84.74)	1	
CMD during pregnancy				
Yes	31 (29.81)	73 (70.19)	2.12 (1.50, 3.00)	1.89 (1.37, 2.61)
No	99 (14.02)	607 (85.98)	1	

^a^Crude relative risk.

^b^Adjusted relative risk adjusted for household income, pregnancy intention, low birth weight, preterm birth, postnatal complications, partner support, history of postnatal common mental disorder before pregnancy, and antenatal common mental disorder.

## 4. Discussion

PCMDs are often overlooked and remain untreated as maternal health conditions. Although nearly one‐third of women in Ethiopia experience these disorders, they are poorly recognized, under‐researched, and receive little governmental attention. This community‐based prospective cohort study screened pregnant women before and after childbirth to assess the prevalence and incidence of common mental disorders during the perinatal period and to identify associated risk factors.

This study found that the prevalence and incidence of PCMDs were 16.05% and 14.02%, respectively, with 3.83% of women experiencing persistent CMD. PCMDs were independently predicted by having a preterm birth, experiencing postnatal complications, and experiencing CMD both before and during childbirth. In this study, 25.06% and 16.05% of women reported symptoms of common perinatal and postnatal CMD, respectively, which aligns with studies conducted in Ethiopia and with the SRMA, which estimated the pooled prevalence of postnatal depression in LMICs [9, 11, 37].

However, the prevalence of PCMDs in this study was lower than that reported in a study conducted in the Amhara Region (33%) [13]. Similarly, the findings of this study reported lower rates of PCMDs compared to those estimated in studies done in Haromaya and Kersa HDSS (24%) [12], as well as in studies conducted in Brazil (20%) [38] and South India (25%) [1]. The potential discrepancy observed may result from differences in the CMD screening tools, cutoff points, study settings, designs, and sample sizes. For example, studies in the Amhara region [13] and the Gurage Zone in Ethiopia [11] used the SRQ‐20 screening tool with a cutoff point of 4/5 to identify postnatal CMD. Additionally, the study in the Gurage Zone evaluated postnatal CMD only among 118 women aged 18–37, which could have led to an overestimation of the prevalence of postnatal CMD.

The possible explanation for the variability in the prevalence of PCMDs among different countries may be due to differences in socio‐cultural and economic factors, as well as the assessment tools employed to evaluate postnatal CMD. For instance, research conducted in Brazil [39] and South India [40] used different tools to assess postnatal CMD, including the Edinburgh Postnatal Depression Scale and the Hospital Anxiety and Depression Scale, respectively. However, the current study utilized SRQ‐20, a culturally adaptable screening instrument developed by the WHO specifically for primary care and community settings, particularly in LMICs [30]. The SRQ‐20 detects psychological distress like anxiety, depression, and somatic symptoms. Variations in heterogeneity and in tool sensitivity and specificity may cause discrepancies in prevalence estimates across studies.

The presence of CMDs before and during pregnancy was a significant predictor of PCMDs, increasing the risk of postnatal CMD by 1.89 and 2.29 times for women who experienced CMD before and during pregnancy, respectively. This finding aligns with studies conducted in Gondar, Northwest Ethiopia [41], Brazil [39], in SRMA conducted in LMICs [24], and in the United States [42], which illustrate how CMD can persist and recur during pregnancy.

Antenatal CMD significantly impacts postnatal mental health through biological and psychological pathways, potentially leading to postnatal CMD [43, 44]. Hormonal imbalances during pregnancy, involving hormones such as estrogen, progesterone, and cortisol, can persist after birth and worsen mental health problems. Fluctuations in these hormones occur throughout pregnancy, and any disturbances can trigger maternal mental health problems [45, 46], which disrupts hormone regulation, affecting maternal stability and resulting in adverse birth outcomes [47–50]. Therefore, early screening during pregnancy is essential to prevent these complications.

This study found that mothers with labor complications and preterm births had a 2.43‐ and 1.72‐times higher risk of PCMD. These results align with studies conducted in the Sodo District, Gondar, Ethiopia, systematic reviews, and Indonesia [41–43, 51]. Self‐reported labor complications, such as traumatic experiences, heavy bleeding, and cesarean section delivery, along with the mother’s perception of her knowledge and negative feelings, can lead to psychological distress during and after pregnancy [44, 45]. Additionally, preterm births are associated with higher rates of posttraumatic stress, with 50% of mothers experiencing PTSD, which increases the risk of PCMD. Furthermore, ongoing chronic stress causes postnatal psychological distress, leading to negative growth and development outcomes [52]. Therefore, it is essential to monitor mothers with a history of labor complications and adverse birth outcomes.

The findings of this study highlight the key connection between antenatal and intrapartum stressors and PCMDs. Postnatal mental health problems are influenced by stress experienced during pregnancy and labor, showing a continuous effect on maternal mental health throughout the postnatal period. This underscores the importance of including mental health screening and counseling in antenatal care and ensuring proper labor management, counseling, and early detection and intervention during pregnancy and childbirth. These measures can enhance postnatal maternal mental health and help reduce the burden of depression and anxiety.

### 4.1. Strengths and Limitations of the Study

The main strengths of this study were its prospective design and large community‐based sample, which made it representative of the prevalence, incidence, and predictors of PCMDs in the Dabat HDSS, Northwest Ethiopia. Using the modified Poisson regression model improved the precision of the estimates. The limitations of this study include reliance on self‐report measures of the history of common mental disorders and labor complications during delivery, which may have introduced recall bias. Paternal mental health and direct measures of household poverty conditions weren’t measured, potentially leading to unobserved confounding. Future studies should assess these factors.

## 5. Conclusions

The incidence and prevalence of postnatal CMD in this study were lower than previously reported in Ethiopia. CMD before and during pregnancy, self‐reported labor complications, and preterm birth were identified as significant predictors of postnatal CMD. Early detection and management of CMD during the perinatal period could prevent maternal complications, postnatal CMD, and adverse effects on infant growth and development. Moreover, enhancing respectful maternity care and involving family members during pregnancy and after birth can reduce maternal anxiety and improve mental health. Improved access to information and counseling during antenatal and intrapartum care further improves maternal well‐being.

## Author Contributions

**Helina Abebe Kurbi:** conceptualization, formal analysis, investigation, methodology, validation, visualization, writing – original draft, writing – review and editing**. Solomon Mekonnen Abebe:** conceptualization, formal analysis, investigation, methodology, validation, visualization, writing – review and editing. **Netsanet Worku Mengistu:** conceptualization, investigation, methodology, validation. **Alemayehu Teklu Toni:** conceptualization, investigation, methodology, validation, writing – review and editing. **Tadesse Awoke Ayele:** conceptualization, formal analysis, investigation, methodology, validation, visualization, writing – review and editing.

## Funding

This research did not receive any specific funding from public, commercial, or not‐for‐profit agencies. However, the University of Gondar provided partial financial support for data collection and had no role in study design, data collection, analysis, manuscript preparation, or the decision to publish.

## Disclosure

The manuscript was edited for language and grammar using Grammarly, and all changes were reviewed and approved by the authors. The authors have read and approved the final manuscript.

## Ethics Statement

This study was reviewed and approved by the Institutional Review Board (IRB) of the University of Gondar with the reference number VP/RTT/Eng./051/61/2021. We also secured approval from the relevant health authorities. All procedures adhered to the Institutional Review Board (IRB) rules and regulations and the 1964 Declaration of Helsinki. Before obtaining written consent, we ensured that all participants were fully informed about the study’s purpose, potential benefits, and risks. Privacy and confidentiality measures were strictly maintained, and participants were notified of their right to withdraw at any time without penalty. The participants were aware that they could withdraw from the study at any point. For participants showing significant symptoms of CMD, our protocol included assessment by mental health professionals and, when necessary, referrals to Gondar University Referral Hospital.

## Conflicts of Interest

The authors declare no conflicts of interest.

## Supporting Information

Additional supporting information can be found online in the Supporting Information section.

## Supporting information


**Supporting Information** The following supporting file is available online in the supporting information section. A completed checklist for the Strengthening the Reporting of Observational Studies in Epidemiology (STROBE) guidelines, indicating where each reporting item is addressed in the manuscript.

## Data Availability

The data that support the findings of this study are available from the corresponding author upon reasonable request.
