# Supplementary material for: Postnatal Common Mental Disorders and Their Predictors in Northwest Ethiopia: A Community‐Based Cohort Study
Source: Depress Anxiety. 2026 May 4;2026:2781884. doi: 10.1155/da/2781884 (PMC13136684; doi:10.1155/da/2781884)
Supplement: Supplementary file 1 — Supporting Information The following supporting file is available online in the supporting information section. A completed checklist for the Strengthening the Reporting of Observational Studies in Epidemiology (STROBE) guidelines, indicating where each reporting item is addressed in the manuscript. [file DA-2026-2781884-s001.docx]

STROBE Statement—Checklist of items that should be included in reports of ***cohort studies***

|  | Item No | Recommendations | Page Number |
| --- | --- | --- | --- |
| **Title and abstract** | 1 | *(*a) Indicate the study’s design with a commonly used term  in the title or the abstract | First page |
|  |  | (*b*) Provide in the abstract an informative and balanced summary of what was done and what was found | Page 1 |
| **Introduction** | | | |
| Background/rationale | 2 | Explain the scientific background and rationale for the investigation being reported. | Page 2-3 |
| Objectives | 3 | State-specific objectives, including any prespecified hypotheses | Page 3 |
| **Methods** | | | |
| Study design | 4 | Present key elements of the study design early in the paper | Page 4 |
| Setting | 5 | Describe the setting, locations, and relevant dates, including periods of recruitment, exposure, follow-up, and data collection. | Page 5-6 |
| Participants | 6 | (*a*) Give the eligibility criteria and the sources and methods of selection of participants. Describe methods of follow-up | Page 5 -6 |
|  |  | (*b*) For matched studies, give the matching criteria and the number of exposed and unexposed |  |
| Variables | 7 | Clearly define all outcomes, exposures, predictors, potential confounders, and effect modifiers. Give diagnostic criteria, if applicable. | Page 5-6 |
| Data sources/ measurement | 8* | For each variable of interest, give sources of data and details of methods of assessment (measurement). Describe the comparability of assessment methods if there is more than one group. | Page 6 |
| Bias | 9 | Describe any efforts to address potential sources of bias | Page 7 |
| Study size | 10 | Explain how the study size was arrived at | Page 6-7 |
| Quantitative variables | 11 | Explain how quantitative variables were handled in the analyses. If applicable, describe which groupings were chosen and why |  |
| Statistical methods | 12 | (*a*) Describe all statistical methods, including those used to control for confounding |  |
|  |  | (*b*) Describe any methods used to examine subgroups and interactions |  |
|  |  | (*c*) Explain how missing data were addressed |  |
| . |  | (*d*) If applicable, explain how loss to follow-up was addressed |  |
|  |  | (*e*) Describe any sensitivity analyses |  |
| **Results** | | | |
| Participants | 13* | (a) Report numbers of individuals at each stage of study—eg, numbers potentially eligible, examined for eligibility, confirmed eligible, included in the study, completing follow-up, and analyzed | Page 13 |
|  |  | (b) Give reasons for non-participation at each stage |  |
|  |  | (c) Consider use of a flow diagram | Page 7-9 |
| Descriptive data | 14* | (a) Give characteristics of study participants (eg, demographic, clinical, social) and information on exposures and potential confounders |  |
|  |  | (b) Indicate the number of participants with missing data for each variable of interest |  |
|  |  | (c) Summarize follow-up time (eg, average and total amount) |  |
| Outcome data | 15* | Report numbers of outcome events or summary measures over time | Page 10 |
| Main results | 16 | (*a*) Give unadjusted estimates and, if applicable, confounder-adjusted estimates and their precision (eg, 95% confidence interval). Make clear which confounders were adjusted for and why they were included | Page 11-11 |
|  |  | (*b*) Report category boundaries when continuous variables were categorized |  |
|  |  | (*c*) If relevant, consider translating estimates of relative risk into absolute risk for a meaningful time period |  |
| Other analyses | 17 | Report other analyses done—eg, analyses of subgroups and interactions, and sensitivity analyses. |  |
| **Discussion** | | | |
| Key results | 18 | Summarize key results with reference to study objectives | Page 11-13 |
| Limitations | 19 | Discuss limitations of the study, taking into account sources of potential bias or imprecision. Discuss both the direction and the magnitude of any possible bias. | Page 15 |
| Interpretation | 20 | Give a cautious overall interpretation of results, considering objectives, limitations, multiplicity of analyses, results from similar studies, and other relevant evidence. | Page 15 |
| Generalizability | 21 | Discuss the generalizability (external validity) of the study results | Page 15 |
| Other information |  |  |  |
| Funding | 22 | Give the source of funding and the role of the funders for the present study and, if applicable, for the original research on which the present article is based. | Page 17 |

|  |  |  |
| --- | --- | --- |
